# A Homodyne Quadrature Laser Interferometer for Micro-Asperity Deformation Analysis

**DOI:** 10.3390/s130100703

**Published:** 2013-01-07

**Authors:** Aljaž PogaČnik, Tomaž Požar, Mitjan Kalin, Janez Možina

**Affiliations:** 1 Iskra Mehanizmi d.o.o., Lipnica 8, Kropa 4245, Slovenia; E-Mail: aljaz.pogacnik@iskra-mehanizmi.si; 2 Faculty of Mechanical Engineering, University of Ljubljana, AškerČeva 6, Ljubljana 1000, Slovenia; E-Mails: tomaz.pozar@fs.uni-lj.si (T.P.); mitjan.kalin@tint.fs.uni-lj.si (M.K.)

**Keywords:** micro-asperity, deformation, real contact area, roughness, polymer, creep, tribology, laser interferometry, homodyne detection, displacement

## Abstract

We report on the successful realization of a contactless, non-perturbing, displacement-measuring system for characterizing the surface roughness of polymer materials used in tribological applications. A single, time-dependent, scalar value, dubbed the collective micro-asperity deformation, is extracted from the normal-displacement measurements of normally loaded polymer samples. The displacement measurements with a sub-nanometer resolution are obtained with a homodyne quadrature laser interferometer. The measured collective micro-asperity deformation is critical for a determination of the real contact area and thus for the realistic contact conditions in tribological applications. The designed measuring system senses both the bulk creep as well as the micro-asperity creep occurring at the roughness peaks. The final results of our experimental measurements are three time-dependent values of the collective micro-asperity deformation for the three selected surface roughnesses. These values can be directly compared to theoretical deformation curves, which can be derived using existing real-contact-area models.

## 1. Introduction

Displacement-measuring interferometers measure linear and angular displacements with nanometer resolution and high accuracy. They are most commonly used for real-time, position-control systems [1] and for the measurements of high-resolution and high-frequency mechanical motions [2]. Even though such interferometers have already reached many research fields and industrial applications, their use as displacement sensors in the field of polymer tribology is rarely encountered. In tribology, due to the loading and friction, two rough mating surfaces are often subjected to deformations with micrometer amplitudes, but nanometer details, such as creep [3]. These deformations require a displacement-measuring sensor with features that call for optical interferometry. This paper presents the application of an interferometer-based measurement system for a specific tribological application.

The use of polymer materials in tribological contacts has increased rapidly in the past few decades, especially because of the low manufacturing costs for mass production, good tribological properties, even without lubrication, lower mass, good damping properties, *etc.* [4–6]. Polymers also have some drawbacks, such as their inferior mechanical and thermal properties, the absorption of water and lower manufacturing tolerances [4–6]. One of the most critical properties of polymer materials is that they can be very sensitive to high temperatures [4,6–9]. Moreover, polymers are viscoelastic materials, *i.e.*, when a polymer is subjected to a constant stress, it experiences a time-dependent deformation, known as polymer creep [3,4]. The above mentioned polymer properties significantly affect the tribological contacts, especially the micro-asperities, which are very difficult to define. However, on the micro-scale, the contact between two rough surfaces occurs on a small number of contacting micro-asperities, *i.e.*, the real contact area, which represents only a fraction of the nominal contact area [10–12]. Misuse of the nominal contact area, where the real contact area should be used instead, leads to an underestimation of the contact pressures and temperatures that occur where two rough surfaces are in contact.

Real-contact-area measurements are, in general, difficult to perform and even the few specific measurement techniques have some limitations, because the small interacting junctions that are involved are hidden (“closed”) within the contact, thus hindering any direct monitoring. The real contact area can be measured with ultrasonic methods [13,14], optical methods [3,15] or with the use of thin films inserted between the contacting surfaces [16]. However, due to the complexity of these measuring methods, theoretical predictions are normally used instead, but the existing theoretical models that predict the real contact area [17–19] also tend to yield values with large uncertainties. This is especially so for polymers, where the sought after values of the real contact area and the corresponding deformations are large because polymers are much softer than metals or ceramics. Therefore, to improve the current predictability, the deformations induced by the loading and the accompanying values of the real contact area need to be understood in greater detail.

The deformation of micro-asperities, which is directly related to the real contact area, can be obtained by monitoring the time-varying behavior of the normal deformation during a mechanical load with a step-like temporal dependence. The expected shape of the deformation history consists of the initial, rapid step that occurs during a smooth application of the load. This step is proportional to the loading in accordance with the linear law of Hooke. It is then followed by a slow increase in the deformation due to a nonlinear creep phenomenon, even though the load remains constant. Intuitively, this slow increase should show some saturation trend, approaching a constant asymptote [4].

Several detection methods can be used to measure such a deformation as a function of time. In general, four basic physical mechanisms can be exploited: piezoelectric, electrostatic, electromagnetic and optical [20]. However, only a few sensors based on the above-mentioned transduction mechanisms feature the following requirements: the detector's output has to be calibrated so that it gives an accurate measurement of the displacement as well as requiring a nanometer resolution. Although the deformation of the micro-asperities is expected to be on a scale of several hundreds of nanometers, the creep process is expected to occur on an even smaller scale, depending on the loading, the size, the roughness and the stiffness of the sample. It also requires a wide dynamic range of at least 10^4^, so that a total displacement of 10 μm can be measured with an accuracy of 1 nm. It must have a flat frequency response from DC up to the maximum frequency expected during the initial application of the load. The detector should also not influence the loading. This precludes the use of sensors demanding direct contact which perturbs the measurement of displacement. Contactless probing, such as with a laser beam, is thus necessary. Finally, the value of the measured displacement must be independent of the load-accompanying acoustic-emission events that release high-frequency, transient, ultrasonic waves [21]. These requirements call for a stand-off and non-perturbing sensor that can be well integrated into the loading mechanism.

We chose an optical detection method that is based on two-beam interference [22]. An optical variation of the classical Michelson interferometer gives two signals in phase quadrature. Such an interferometer, called a homodyne quadrature laser interferometer (HQLI) [23–25], features constant sensitivity with a sub-nanometer resolution, a wide dynamic range and a flat frequency response. Alternative interferometric techniques, such as other variants of homodyne interferometry [26–28], heterodyne interferometry [29] and low-cost feedback interferometry [30], may also be employed to measure the collective micro-asperity deformation. Non-interferometric optical methods, such as the laser-beam deflection technique [31], which is also employed in atomic force microscopy to measure the deflections of the cantilever, can be used as well. Compared to the above-mentioned interferometric alternatives, HQLI has a simple electronic and optical construction, where all of the components can be easily available on market for a reasonable price. Its linearity is better compared to the heterodyne interferometers and it has a better resolution compared to the feedback interferometers. It main drawback is related to homodyne detection technique which is susceptible to the amount of light return and laser power fluctuations.

In this paper we report on the realization of the measuring procedure to determine the proposed time-dependent, collective micro-asperity deformation of a rough polymer surface. We employed a HQLI to measure the displacements as it provides the accuracy and repeatability required for measurements of the collective micro-asperity deformation on the nanoscale. Throughout the paper, the required steps to obtain the value of the collective micro-asperity deformation measurements are described in detail. The collective micro-asperity deformations for three different polymer surface roughnesses (*R_a_* = 0.40 μm, *R_a_* = 1.5 μm and *R_a_* = 2.5 μm) were measured at a normal load of 390 N, corresponding to a nominal contact pressure of 5.0 MPa.

## 2. Experimental Setup

All the symbols and abbreviations used in the paper are gathered in Table 1 in the Appendix.

### 2.1. Sample Preparation

The material used for the measurements of the collective micro-asperity deformations was an unreinforced polyamide 6 (PA6, Ultramid^®^ B3S, BASF, Ludwigshafen, Germany). The raw material was produced in granular form and then injection molded to produce the desired cylindrical form. Due to the uneven shrinkage of the material, the polymer specimens were additionally machined so that the top and bottom surfaces were parallel. The finalized samples were 10.0 mm high and 10.0 mm in diameter.

The different roughnesses were obtained with a sequence of grinding and polishing steps to achieve the three selected surface roughnesses. The surface-roughness parameters were measured using a stylus-tip profiler (T8000, Hommelwerke GmbH, Schwenningen, Germany). The surface roughnesses of the samples were *R_a_* = 0.40 μm, *R_a_* = 1.50 μm and *R_a_* = 2.50 μm. The reference surface, referred to as the smooth surface, was polished as smoothly as possible with our polishing apparatus. Its measured roughness was *R_a_* = 0.08 μm.

### 2.2. Measuring Procedure

There are several things one needs to address when measuring the collective micro-asperity deformation of polymer materials. When a polymer material is compressed, several different deformations add up to the final deformation of the sample. The deformation of the polymer sample occurs due to the compression loading. As a result of polymer creep, a time-dependent component of the deformation is also present. And when measuring rough surfaces, the sum of the individual micro-asperity deformations also adds up to the total deformation of the polymer sample.

Besides the above-mentioned deformations, our measuring system also senses the mechanical deformations and vibrations of the whole experimental setup as well as the whole body motion of the sample. As we were only interested in the collective micro-asperity deformation, the following measuring procedure was used.

**Figure 1.**
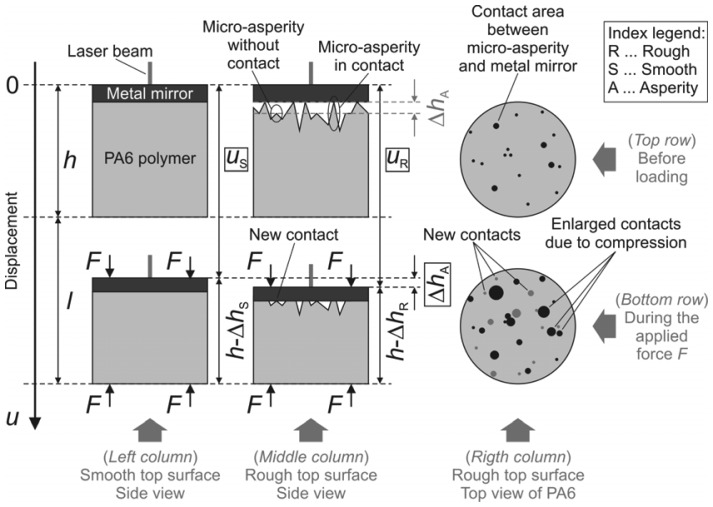
Measuring geometry at two time instances.

The measuring geometry of the samples at two time instances is presented in Figure 1. The top row shows the polymer sample with exaggerated micro-asperities and the metal mirror before loading, while the bottom row depicts a similar measuring geometry during the applied normal load *F*. A side view of the smooth sample is shown in the left-hand column, while the rough one is sketched in the middle column. The right-hand column shows the top view of the rough sample.

The dots of various sizes represent the contact areas between the rough top surface of the polymer and the bottom surface of the metal mirror before loading (top row) and during loading (bottom row). In practice, these areas have irregular shapes even though they are shown as circles. The size of the initial micro-asperity contact areas (the black spots) increases when the load is applied. In addition, the new contact areas (the grey spots) show up as the load gets larger, because the mirror deforms the initial micro-asperities and comes into contact with those that were initially slightly below the bottom surface of the metal mirror.

The top specular surface of the uncompressed metal mirror is initially set to zero displacement. The total height of the polymer sample and the metal mirror is denoted as *h*, while *l* corresponds to the distance between the bottom part of the polymer before and during the loading. The value of *l* is non-zero, because the loading also slightly deforms the grounding on which the polymer is placed. For presentation purposes in Figure 1 the distance *l* is intentionally exaggerated. In reality, the value of *l* is expected to be much smaller than *h* (*l* ≪ *h*). The interferometer measures the distance *u* traveled by the top surface of the metal mirror during the normal loading. Figure 1 also shows the probing laser beam that encodes the displacement *u* into an optical phase *p*. Due to the compressive loading, the total height *h* is reduced by Δ*h*. When the contribution of the micro-asperity deformations to Δ*h* is negligible, such as when smooth samples are used, Δ*h* corresponds to the contraction of the bulk medium. The difference between the absolute contraction of the rough polymer sample Δ*h*_R_ and the smooth sample Δ*h*_S_ is the sought value for the collective micro-asperity deformation Δ*h*_A_.

**Figure 2.**
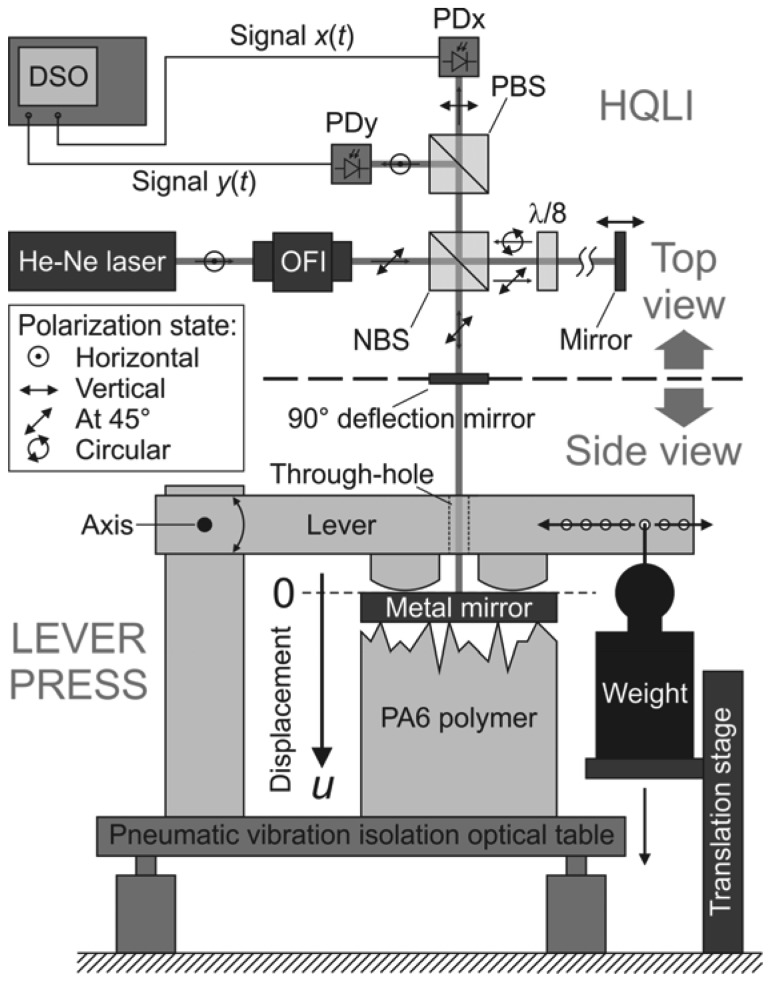
Optical setup of the homodyne quadrature laser interferometer (HQLI) and a schematic view of the lever press.

To obtain Δ*h*_A_ experimentally, two displacement measurements had to be performed. First, a smooth (*R_a_* = 0.08 μm) polymer sample was mounted into the lever press (see Figure 2). Then the normal load was applied and the time-dependent total displacement of the smooth sample *u*_S_(*t*) was measured with the HQLI. The same sample was then removed from the lever press. Its smooth surface was re-grinded to one of the three selected surface roughnesses (*R_a_* = 0.40 μm, *R_a_* = 1.50 μm or *R_a_* = 2.50 μm). Then, the re-ground polymer cylinder was reinserted into the lever press and the displacement measurement of the rough sample *u*_R_(*t*) was acquired again. The loading conditions were unchanged. A total of six displacement measurements had to be realized to obtain the time-dependent, collective micro-asperity deformations for the three surface roughnesses.

For idealized conditions, the following equalities can be geometrically derived from Figure 1:

(1) $$u_{R}+\left(h-\Delta h_{R}\right)=h+l\;\text{and}\;u_{S}+\left(h-\Delta h_{S}\right)=h+l.$$

All the quantities in Equation (1), except *h*, are time dependent. The ideal displacement of the rough *u*_R_ and the smooth *u*_S_ sample is:

(2) $$u_{R}=l+\Delta h_{R}\;\text{and}\;u_{S}=l+\Delta h_{S}.$$

Combining both ideal interferometric measurements one obtains:

(3) $$u_{R}-u_{S}=\Delta h_{A}=\Delta h_{R}-\Delta h_{S}.$$

### 2.3. Measuring Uncertainties

In contrast to the ideal measurement of Δ*h*_A_ [Equations (1)–(3)], the real measurement is hampered by the time-dependent measuring uncertainty *δh*_A_:

(4) $$u_{R}-u_{S}=\Delta h_{A}+\delta h_{A}.$$

This uncertainty depends on the following six terms:

(5) $$\begin{array}{cc} \delta h_{A} & =\delta l+\delta u_{\text{Env}}+\delta u_{\text{AE}}+\delta u_{\text{Air}}+\delta u_{\cos}+\delta u_{\text{Noise}}\\ & =\left(l_{R}-l_{S}\right)+\left(u_{\text{Env},R}-u_{\text{Env},S}\right)+\left(u_{\text{AE},R}-u_{\text{AE},S}\right)\\ & +(u_{\text{Air},R}-u_{\text{Air},S})+(u_{\cos,R}-u_{\cos,S})+\left(u_{\text{Noise},R}-u_{\text{Noise},S}\right) \end{array}$$

The distances appearing in the first term, *l*_R_ and *l*_S_, are practically equal. It is expected that they are only dependent on the instantaneous value of the normal loading force *F*. Their difference *δl* cannot be determined with the HQLI, because it is not possible to separate the contributions of *l* and Δ*h* with a single measurement of *u* [see Equation (2)].

The second term describes the total contribution to the uncertainty from environmental effects. Among the most prominent are the low-frequency mechanical vibrations, the sound waves and the air flow in the interferometer. The mechanical vibrations can be significantly reduced by isolating the optical table, which supports the experimental setup, from the floor of the room.

Acoustic emission accompanies the rapid inelastic events when the micro-asperities are deformed due to the applied pressure. Such inelastic events release high-frequency mechanical waves, *i.e.*, ultrasound, that propagate from the asperities to the top of the metal mirror. Their reflection from the top surface of the mirror results in the displacement of the surface. The normal component of the ultrasound-caused displacement thus corrupts the measurement of Δ*h*_A_. The problem arises when the displacement amplitude of the ultrasound exceeds *λ*/4, which may lead to an *mλ*/2 offset in *u* and consequently in Δ*h*_A_. Here, *λ* is the laser wavelength in air, *m* is an integer number and the offset originates from the phase unwrapping.

The fourth term deals with the influence of the refractive index of the air. Its relative uncertainty *δu*_Air_/Δ*h*_A_ equals the relative uncertainty of the compensated refractive index of the air *δn*/*n*.

The cosine error, represented by the fifth term, is a geometrical error that results from an angular misalignment between the measurement beam and the axis of motion. This occurs when the measuring surface experiences a tilt away from its normal motion. Homogeneous loading of the sample minimizes this error. Since in our case, the axis of measurement is not offset from the axis of interest, there is no Abbé error present.

The last term in Equation (5) represents the noise. The minimal detectable displacement of any displacement-measuring sensor, defined as the value corresponding to a signal-to-noise ratio of unity, is set by the fundamental limit called the thermal rattle or *phonon* shot noise [32]. However, all optical detection techniques, regardless of whether they are interferometric or non-interferometric, have a much larger minimum detectable displacement, which is given by the *photon* shot noise [23]. The amplitude of this noise is about two orders of magnitude larger than the phonon noise [32]. The limiting resolution of the detected displacement due to the quantum laser amplitude noise (the photon shot noise) is independent of the frequency; it dominates the detector noise if the interferometric laser output power *P*_L_ is around 1 mW, and has a value of:

(6) $$4.5\times 10^{-17}\sqrt{\frac{\Delta f}{P_{L}}}\text{m}\sqrt{J}$$

where Δ*f* is the measuring frequency bandwidth of the optical sensor.

Often, discretization of the analogue signal by an *n*-bit analogue-to-digital converter (ADC) sets an even larger practical minimum detectable displacement. In our single-pass interferometer, the peak-to-peak amplitude of the interference signal corresponds to a *λ*/4 displacement. This value is then ideally further subdivided 2*^n^*-times by an ADC into *λ*/(4 × 2*^n^*)-large partitions. A single partition is the displacement unit, the practical minimum detectable displacement, set by the analogue-to-digital conversion part of the detection chain. Since in practice the peak-to-peak amplitude of the interference signal does not fit perfectly to the operating range of the ADC, the practical minimum detectable displacement is even larger than *λ*/(4 × 2*^n^*) by a factor of the ratio between the full span of the ADC and the peak-to-peak amplitude of the signal.

### 2.4. Construction of the HQLI and the Lever Press

The optical setup of the homodyne quadrature laser interferometer (HQLI) and the mechanical parts of the lever press are sketched in Figure 2. The HQLI is shown in the top-view perspective, while the level press is shown from the side. All the components shown in Figure 2 are placed on the optical table, except for the digital sampling oscilloscope (DSO) and the linear translation stage.

The interferometer uses a stabilized (vacuum wavelength *λ*_v_ = (632.9914 ± 0.0003) nm; amplitude stability over 1 min < 0.2% and amplitude noise (0–10 MHz) < 0.2%) and linearly polarized He-Ne laser (Model SL 02/1, SIOS Meβtechnik GmbH, Ilmenau, Germany). The polarization plane of the exiting beam with power *P*_L_ = 1 mW, 1/e^2^-diameter of 0.63 mm and full-angle divergence of 1.3 mrad is perpendicular to the optical table (vertical polarization state). Once the beam passes through the optical Faraday isolator (OFI), its polarization plane is rotated by 45 degrees. The beam is then equally distributed into two arms of the interferometer by a nonpolarizing beam splitter (NBS). The transmitted beam travels in the reference arm and is reflected by the static mirror. It passes through the octadic wave plate (*λ*/8) twice before it re-enters the NBS. Since the octadic wave plate has its fast axis perpendicular to the optical table it induces a 90-degree phase shift between the horizontal and vertical polarization components of the reference beam. Equivalently, a double passage of the beam in the reference arm through the octadic wave plate transforms its linear polarization into the circular form. The beam that is deflected by the NBS enters the measuring arm of the HQLI. First, it is parallel with the optical table. The deflection mirror is then used to direct the beam perpendicularly to the metal mirror placed on top of the polymer. When the measuring beam is reflected from the metal mirror, the motion of its top specular surface *u* along the beam is encoded in the optical phase *p*. The measuring beam is reflected from the center of the metal mirror, where it has a diameter of 1.2 mm. The linearly polarized measuring beam then returns to the NBS, where it is recombined with the circularly polarized beam returning from the reference arm. Due to coherence and wavefront-superposition problems, both arms have to be of about the same length. The construction of the press demanded a fixed length for the measuring arm, which was set to 25 cm, measured from the center of the NBS to the top surface of the metal mirror. The mirror in the reference arm was translated to match the length of the measuring arm in order to maximize the contrast of the interference signal.

Two compound beams then leave the NBS. One returns toward the laser and is blocked by the OFI, which prevents optical feedback and possible destabilizations of the laser. The other one heads toward the polarizing beam splitter (PBS), which transmits the horizontal polarization and reflects the vertical polarization. The homemade, DC-200 MHz, pre-amplified photodiodes PDx and PDy measure the power of the interfering light. If the metal mirror experiences a uniform motion *u*, the power detected by each photodiode changes harmonically with a period *λ*/2. However, due to the phase shift induced by the octadic wave plate, the detected powers are in quadrature, *i.e.*, 90 degree out of phase. The signals *x* and *y* are the preamplified output signals of the photodiodes PDx and PDy, respectively. They are acquired and digitalized by an 8-bit DSO (500 MHz WaveRunner 6050A, LeCroy, New York, NY, USA). Once the raw signals are acquired from the photodiodes, the rest of the data processing is done offline.

The lever press is used to apply a normal load to the metal-mirror-polymer contact. The lever arm is fixed to the axis on one side, which allows the lever arm to rotate around the axis. Above the center position of the metal mirror, a through-hole was drilled to allow the laser beam to reach the reflecting surface of the metal mirror. On the other side, a dead weight is mounted on to the lever. The distance from the place where the weight is hung to the axis can be adjusted in order to obtain the desired normal force on the metal mirror. A 390-N normal force is used for the measurements, which corresponds to 5.0 MPa of nominal contact pressure. A motor-driven, linear translation stage is used to lower the weight during the loading. In the initial position there was no contact between the lever arm and the top surface of the mirror. However, once the smooth loading began, the lever came into contact with the mirror. The applied normal force increased linearly until the total weight was transferred to the lever. At this point the translation stage lost contact with the weight and the desired maximum load was achieved. The HQLI and the lever press are mounted on the pneumatic vibration-isolation table in order to minimize the environmental vibrations. The translation stage was intentionally mounted separately from the vibration-isolation table to minimize the influence of the motor-generated vibrations on the measurement.

### 2.5. Signal Processing

**Figure 3.**
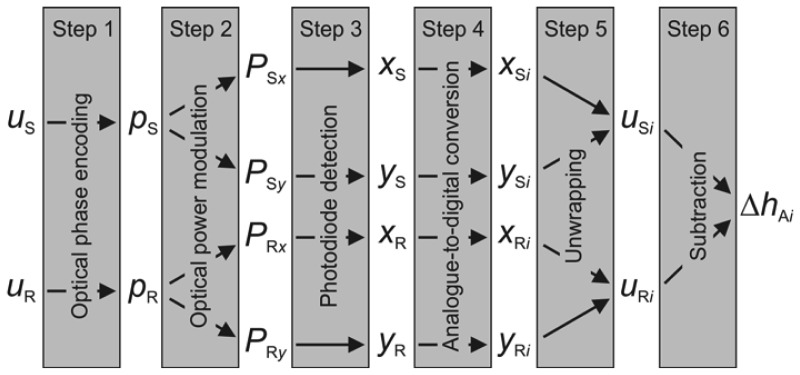
Block diagram of the signal processing.

We will describe the necessary steps, presented in the block diagram in Figure 3, that have to be performed to obtain the discrete dataset of the time-varying, collective micro-asperity deformation Δ*h*_A_*_i_*(*t*). The input parameters enter each block from the left-hand side, while the output parameters exit the block on the right-hand side.

Interferometric detection with the HQLI consists of the following steps. First, the normal displacement *u*(*t*) is converted into the optical phase:

(7) $$p(t)=\frac{4\pi n}{\lambda_{v}}u(t)=\frac{4\pi }{\lambda }u(t)$$

of the measuring beam, where *n* is the refractive index of the air in both arms of the interferometer. Second, the optical phase modulation of the measuring beam is transformed into an optical power modulation with the help of the interference between the measuring and the reference beam. Third, the optical power reaching both photodiodes is converted into amplified voltage signals *x*(*t*) and *y*(*t*), which, ideally, have the following form:

(8) $$x(t)=\frac{V_{0}}{4}\left(1+\sin p(t)\right)\;\text{and}\;y(t)=\frac{V_{0}}{4}\left(1+\cos p(t)\right)$$

Here, *V*_0_ is the output photodiode voltage if the whole output interferometric beam was collected by a single photodiode. A detailed treatment of the first three steps can be found in [25].

The fourth step consists of the digitalization of *x*, *y* and *t*. The analogue signals *x* and *y* were equidistantly sampled by the 8-bit DSO with a sampling capacity of 1 MS (mega sample) per channel. Due to the discretization, the ideal practical minimum detectable displacement is *λ*/(4 × 2^8^) = 0.6 nm, but since the peak-to-peak amplitude 2*V*_0_ does not fit perfectly with the operating range of the DSO's ADC, the minimum detectable amplitude is slightly larger, but remains below 1 nm. An external trigger starts the acquisition of the *x* and *y* and sets the time to 0. After about 8 s, the linear translation stage begins its uniform motion. The acquired discrete datasets [*x_i_* = *x*(*t_i_*), *y_i_* = *y*(*t_i_*), *t_i_*] for *i* = 1,…,10^6^ are then processed offline. The successive data points of a measurement lasting 200 s are thus separated in time by Δ*t* = *t_i_*_+1_ − *t_i_* = 0.2 ms. In our case, sampling of the DSO significantly reduces the 200-MHz upper frequency bound of the interferometer's output and sets it to 1/(2Δ*t*) = 2.5 kHz, according to the Nyquist sampling theorem. Higher sampling rates could bring the upper frequency bound of the whole detection chain (2.5 kHz) much closer to the upper bound of the interferometer (200 MHz).

In the fifth step, the displacement *u_i_* is obtained by using the basic unwrapping Equation [25]:

(9) $$u_{i}=\frac{\lambda_{v}}{4\pi n}\left[\arctan\left(\frac{x_{i}-V_{0}/4}{y_{i}-V_{0}/4}\right)+m\pi \right],m=0,\pm 1,\pm 2,...$$

The integer *m* has to be chosen correctly so that the differences between the successive values of the displacement *u_i_*_+1_ − *u_i_* are smaller than *λ*/4. This condition requires that the measured velocity (*u_i_*_+1_ − *u_i_*)/Δ*t* does not exceed *λ*/(4Δ*t*) = 0.8 mm/s, further reducing the upper frequency bound of the detection bandwidth to 1.25 kHz. For the sake of conciseness, the fourth and the fifth steps were presented only for the ideal case.

However, the experimentally obtainable signals *x* and *y* are corrupted by the so-called nonlinearities [33–36], which have many origins. The most prominent one is attributed to imperfect optical components. For this reason, the corrupted signals differ slightly from those given in Equation (8). When they are inserted into the basic unwrapping Equation (9), the measured displacement deviates from the true displacement by a periodic error. In order to effectively reduce this error, a scale linearization must be performed. Our scale-linearization technique employs a more general phase-unwrapping digital computation described in detail in [37]. This computation is based on an enhanced ellipse fitting that determines the most common nonlinearities, *i.e.*, the unequal gain of both signals, their non-zero offsets and the phase-shift error, and then corrects them. Apart from these periodic errors, the scale linearization also addresses, to a certain extent, the noise component on the quadrature signals, ADC nonlinearities and sampling error. In the final, sixth step, the collective micro-asperity deformation Δ*h*_A_*_i_* is calculated simply as a point-by-point subtraction of *u*_S_*_i_* from *u*_R_*_i_*.

To summarize, the presented HQLI has the following features. It measures only the out-of-plane component of the surface displacement. Its resolution is below 1 nm. Its optical part including the photodiodes and their preamplifiers has a flat frequency response from DC up to 200 MHz (−3 dB). Its sensitivity is constant at a value of 12 mV/nm for full light return from the measuring surface. Its dynamic range is defined as the total displacement achievable with a given resolution, divided by the resolution. It is thus primarily determined by the minimum detectable displacement, the basic resolution, which is below 1 nm, and by the maximum displacement that can be measured with an uncertainty equal to the basic resolution. The latter physical bound is given mainly by the accuracy of the calculated value of the refractive index of the air *n* and by its variation *δn* during the time of measurement.

## 3. Results and Discussion

**Figure 4.**
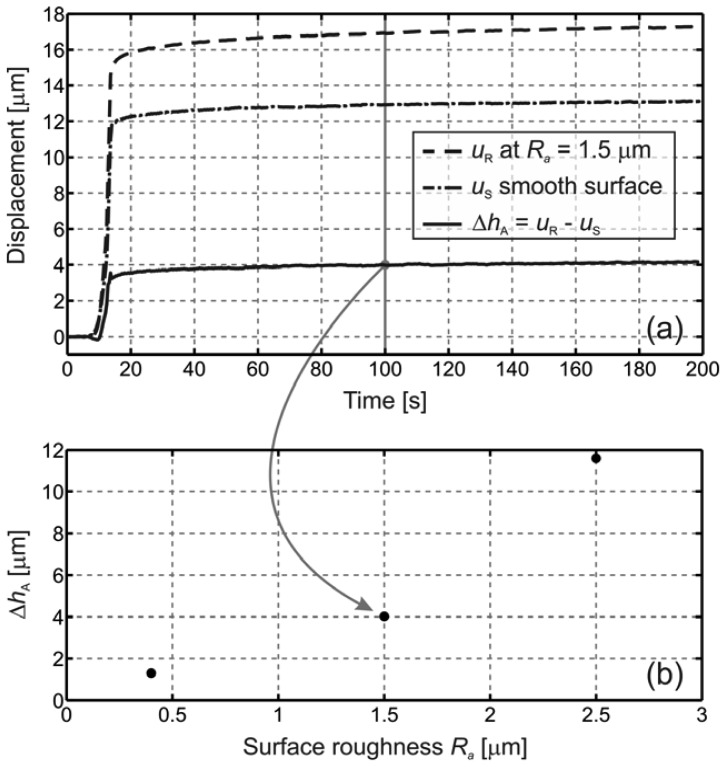
(**a**) Displacement history curves for the smooth surface *u*_S_, for the rough surface *u*_R_ of *R_a_* = 1.5 μm, and for the corresponding collective micro-asperity deformation Δ*h*_A_. (**b**) Collective micro-asperity deformations for three different surface roughnesses.

Figure 4(a) shows the measured displacements of *u*_R_ (sample surface roughness of *R_a_* = 1.50 μm) and *u*_S_ (smooth reference surface) as a function of time for the 200-s duration of the measurement. Their difference, the collective micro-asperity deformation Δ*h*_A_, is also presented. The loading began 8 s after triggering. The linear loading process lasts approximately 5 s until the maximum force of *F* = 390 N is reached and is thereafter kept constant. When the maximum force was reached, the value of the displacement *u*_S_ was around 12.0 μm and that of the displacement *u*_R_ was about 15.0 μm. It is clear from Figure 4(a) that once the maximum force was achieved and left unchanged, the displacements *u*_S_ and *u*_R_ continue to increase. In the subsequent 3-minute interval, the displacement *u*_S_ is increased by 1.2 μm, and *u*_R_ by 2.0 μm. The point-by-point subtraction of *u*_S_ from *u*_R_ yields the collective micro-asperity deformation curve Δ*h*_A_. All three curves, *u*_S_, *u*_R_ and Δ*h*_A_, show some saturation trend. The slope of the creep gets smaller with time and approaches a horizontal asymptote. The collective micro-asperity deformation of the bottom smooth surface (*R_a_* = 0.08 μm) of the PA6 had a negligible contribution to the Δ*h*_A_.

The value of Δ*h*_A_ at 100 s [the solid vertical grey line in Figure 4(a)] is shown as the middle measured point in Figure 4(b). In addition, similar results for the surface roughnesses *R_a_* = 0.40 μm and *R_a_* = 2.50 μm are presented in Figure 4(b). The collective micro-asperity deformation increases with increasing surface roughness.

The initial step in the displacements *u*_S_ and *u*_R_, which occurs in the time interval between 8 s and 13 s, involves two terms: *l* and Δ*h* [see Equation (2)]. The distance *l* between the bottom part of the polymer before and during loading is dependent merely on *F*. The second term, Δ*h*, corresponds to mutual contractions of the elastic metal mirror and the viscoelastic polymer. In the subsequent 3-minute interval, the absolute contraction of the metal mirror remains constant, but the contraction of the polymer increases, even though the load remains constant. This is a clear indication of polymer creep. The creep is present for both smooth and rough surfaces; however, the creep is more pronounced for rough surfaces, which can be seen from the curve of Δ*h*_A_. This brings us to a conclusion, that even though the creep of the bulk polymer material is subtracted, the remaining creep is the result of the collective micro-asperity creep. It is also seen that the value of the creep at *t* = 100 s is in the range of 100 nm, confirming the need for nm-scale resolution.

As the weight is lowered by the linear translation stage that always moves uniformly with the same constant velocity, the weight is detached from the translation stage sooner for the sample with the smooth surface than for the one with the rough surface, because the initial displacement step for *u*_R_ is larger than for *u*_S_. The sooner the weight is detached, the sooner the maximum normal load is achieved. This implies that the shape of Δ*h*_A_ should differ in shape compared to either *u*_R_ or *u*_S_ during the application time of the load. For the above-mentioned reasons, some deviations of the Δ*h*_A_ curve compared to the *u*_R_ and *u*_S_ curves are expected. The most obvious ones are the depression and the peak of Δ*h*_A_, at the beginning and at the end of the loading step, respectively.

The six contributions to the uncertainty of the measured, collective micro-asperity deformation *δh*_A_ that are gathered in Equation (5) will now be briefly assessed.

The first term, *δl*, is significant only during the time period corresponding to the loading step between *F* = 0 N and *F* = 390 N, because *l* depends only on *F* and the shape of *F*(*t*) differs when measuring *u*_S_ and *u*_R_ only during the loading step. For all the other times, the contribution of *δl* to *δh*_A_ is negligible, especially compared to the second term.

Of the environmental effects contained in the second term of Equation (5), the mechanical vibrations are the most pronounced. Their frequency content is below 1 kHz and can easily be followed by the HQLI. We found that their amplitude can be as large as 50 nm if no attempt is made to dampen them. When two vibration-corrupted displacement curves are subtracted, the uncertainty can, in the worst-case scenario, double to a value of 100 nm. However, when we activated the pneumatic isolation of the optical table, the maximum amplitude of the environmental vibrations decreased to 10 nm, resulting in a maximum error of 20 nm. This term is the largest of the six terms and contributes the most to the final measurement uncertainty.

Acoustic emission (AE) transients, described by the third term, that are composed of high frequencies over 10 kHz are unnoticed during the measurement, due to the low sampling frequency. However, an *mλ*/2 error in Δ*h*_A_ may occur during the unwrapping process as a result of possible, but improbable, high-frequency and high-amplitude AE events.

The fourth term can be evaluated with the help of Equations (7) and (9). Since the relative uncertainty of the compensated refractive index of the air *δn*/*n* under normal laboratory conditions is below 10^−6^, the same value of the relative uncertainty is expected for *δu*_Air_/Δ*h*_A_. In fact, this value equals the inverse of the dynamic range. The refractive index *n* depends predominantly on the following air conditions in our laboratory: air temperature, the actual local atmospheric pressure that is not reduced to sea level, the relative air humidity, and the carbon dioxide content [37]. These four parameters were input into Ciddor empirical equation [38] to evaluate *n*. The values of the first three parameters were measured before each measurement of displacement and we used a fixed value of 450 ppm, which is about 60 ppm above the outdoor value in the year 2012. The vacuum wavelength *λ*_v_ has to be divided by the resulting index of refraction of the air to obtain the laser wavelength in air *λ* = *λ*_v_/*n*. This value was used in the evaluation of Equation (9). If the typical conditions were changed in the temperature by 1 °C or in the pressure by 0.37 kPa or by 90% in the relative humidity, the relative variation of *n*, *δn*/*n*, would change by about 10^−6^ (1 ppm). The variation of the temperature, pressure and humidity during our measurement of the displacement, as well as the uncertainties of our measurement of these three quantities, are always much smaller than the values that correspond to a change of 1 ppm. Because we made the compensation for the environmental effects on *n*, the dynamic range of our HQLI is larger than 1/(1 ppm) = 10^6^.

The cosine error due to the relative tilt of the metal mirror with respect to the top rough surface of the PA6 was also assessed. The largest measured degradation of the contrast was around 20%. If this reduction of contrast is solely attributed to the tilt of the mirror, and thus to an angular displacement between the interfering beams, the angle of the tilt is calculated to be smaller than 0.34 mrad. This yields a negligible relative uncertainty of 6 × 10^−8^ due to the cosine error. Since the measurements were performed in the center of the metal mirror, using the above value for the angle of the maximum tilt, the edges could deviate from the middle point in the worst case by ±1.7 μm. However, this assessment is exaggerated, because the construction of the lever press prevents uneven loading near the rim of the metal mirror, and such large deviations are unlikely to take place.

Finally, the noise of our detection system, given by the sixth term, is well below the discretization quantum of about 1 nm.

The contributions of the first and second terms to the total uncertainty of the measured, collective micro-asperity deformation *δh*_A_ can be fully eliminated by a special, differential design of the current HQLI. The time stability of the HQLI was also measured. The temperature drift of the interferometer was measured several times during the day. We found that during a 200-s measurement the temperature drift in the laboratory was never larger than ±0.01 °C and the corresponding drift of the interferometer was never larger than ±2 nm. The temperature vs. time curve of the temperature sensor, which measured the temperature of the air near the NBS, and the “displacement”-drift curve, had an equal shape with either the same polarity or with an inverted polarity, which proves that the drift of the interferometer is caused by the drift of the temperature.

The total measured root-mean-square uncertainty of Δ*h*_A_ is 6 nm. This uncertainty was obtained without a loading on the activated pneumatic isolation of environmental vibrations. It includes the second term in Equation (5), excludes the first and third terms in Equation (5) and takes into account the thermal drift. During the measurement of this uncertainty, all the moving parts of the experiment were running: the fan in the DSO and the linear translations stage.

Based on the performed measurements and the analysis of the measuring uncertainty, the presented measuring system for the determination of the collective micro-asperity deformation can be used even when the roughness and the height of the polymer sample is scaled down. It can measure similar deformations, even when thinner, less rough and less compressed samples with larger Young's moduli are used, especially when a differential optical arrangement is employed.

## 4. Conclusions

The presented research was motivated by the rapid increase in the use of polymer materials for tribology due to their advantages over metallic and ceramic materials. We provide a detailed description of an experimental system that was specially developed to measure the surface roughness of polymer materials used in tribological applications. The described experimental system consists of two main parts: the displacement-measuring homodyne quadrature laser interferometer (HQLI) and the lever press.

The HQLI measures only the out-of-plane component of the surface displacement. It has a resolution of 0.6 nm, a flat frequency response from DC to 1.25 kHz, a constant sensitivity of 12 mV/nm and a wide dynamic range of about 10^6^.

Subtraction of the displacement obtained using a polymer with a smooth surface from the one with a rough surface gave a time-dependent, single scalar quantity called the collective micro-asperity deformation. The measurement of this parameter was proposed, because it can be directly compared to theoretical deformation curves, which can be derived using existing real-contact-area models.

The results of the experimental measurements are three collective micro-asperity deformation histories obtained for three different surface roughnesses (*R_a_* = 0.40 μm, *R_a_* = 1.50 μm and *R_a_* = 2.50 μm) that were acquired during the step-like loading, whose maximum nominal contact pressure equals 5.0 MPa.

The measurements led us to the following conclusions. The collective micro-asperity deformations for all three PA6 cylindrical samples (10.0 mm in height and 10.0 mm in diameter) are on a scale of several hundreds of nanometers. This deformation is larger with higher top-surface roughnesses, which is consistent with the preliminary results given by the existing theoretical models. The polymer-creep phenomenon is detected not only for the bulk polymer, but has a contribution that originates solely from the micro-asperity creep and has an amplitude of about 100 nm. The measurements also indicate that as time passes, while the maximum loading force is kept unchanged, the displacements approach a horizontal asymptote, which sets the maximum displacement value for a given loading.

The physical characteristics of the measuring system also enable measurements of thinner samples that have smaller surface roughnesses, may be subjected to smaller loads and could be of a stiffer material, which all results in smaller, collective micro-asperity deformations.

## Appendix

**Table 1.** Nomenclature.

**Symbol**	**Description**	**Units**
*u*	Out-of-plane displacement	m
*h*	Distance from the top of the metal mirror to the bottom of the PA6 polymer (uncompressed)	m
Δ*h*	Absolute contraction of *h* due to loading	m
Δ*h*_A_	Collective micro-asperity deformation	m
*l*	Distance between the bottom part of the PA6 polymer before and during loading	m
*R_a_*	Arithmetic average of the absolute values of the surface profile	m
*λ*	Wavelength of the He-Ne laser beam in air under standard conditions	m
*λ*_v_	Vacuum wavelength of the He-Ne laser beam	m
*F*	Loading force	N
*t*	Time	s
Δ*t*	Sampling period	s
*x*	Output signal from the photodiode PDx	V
*y*	Output signal from the photodiode PDy	V
*V*_0_	Photodiode output voltage when it collects the power *P*_L_	V
*P*_L_	Laser output power	W
*P*	Laser beam power	W
Δ*f*	Frequency bandwidth of the detector	Hz
*i, n*	Positive integer	1
*n*	The refractive index of the air	1
*m*	Integers	1
*p*	Optical phase	rad

**Abbrev.**	**Description**	

S	Smooth	
R	Rough	
A	Asperity	
V	Vacuum	
PA6	Polyamide 6 polymer	
PTFE	Polytetrafluoroethylene (Teflon)	
ADC	Analogue-to-digital converter	
DC	Direct current (zero frequency)	
HQLI	Homodyne quadrature laser interferometer	
DSO	Digital sampling oscilloscope	
PDx	Photodiode measuring the horizontally polarized light	
PDy	Photodiode measuring the vertically polarized light	
PBS	Polarizing beam splitter	
NBS	Nonpolarizing beam splitter	
*δ*	Uncertainty	
*λ*/8	Octadic wave plate	
OFI	Optical Faraday isolator	
AE	Acoustic emission	
Env	Environmental	

## References

[1] Manske E., Jäger G., Hausotte T., Füβl R. (2012). Recent developments and challenges of nanopositioning and nanomeasuring technology. Meas. Sci. Technol..

[2] Požar T., GregorČiČ P., Možina J. (2009). Optical measurements of the laser-induced ultrasonic waves on moving objects. Opt. Express.

[3] Ovcharenko A., Halperin G., Etsion I. (2009). Experimental Study of a Creeping Polymer Sphere in Contact With a Rigid Flat. J. Tribol..

[4] Strong A.B. (2006). Plastics: Materials and Processing.

[5] Erhard G. (2006). Designing with Plastics.

[6] Budinski K.G., Budinski M.K. (2009). Engineering Materials: Properties and Selection.

[7] Harper C. (2006). Handbook of Plastics Technologies: The Complete Guide to Properties and Performance.

[8] PogaČnik A., Kalin M. (2012). Parameters influencing the running-in and long-term tribological behaviour of polyamide (PA) against polyacetal (POM) and steel. Wear.

[9] Computer Aided Material Preselection by Uniform Standards (CAMPUS) http://www.campusplastics.com/.

[10] Bhushan B. (2002). Introduction to Tribology.

[11] Stachowiak G.W., Batchelor A.W. (2005). Engineering Tribology.

[12] McLaskey G.C., Glaser S.D. (2011). Micromechanics of asperity rupture during laboratory stick slip experiments. Geophys. Res. Lett..

[13] Pau M. (2003). Estimation of real contact area in a wheel-rail system by means of ultrasonic waves. Tribol. Int..

[14] Gonzalez-Valadez M., Dwyer-Joyce R.S. (2009). Asperity Creep Measured by the Reflection of Ultrasound at Rough Surface Contact. J. Tribol..

[15] Ovcharenko A., Halperin G., Etsion I., Varenberg M. (2006). A novel test rig for in situ and real time optical measurement of the contact area evolution during pre-sliding of a spherical contact. Tribol. Lett..

[16] Sick J.-H., Ostermeyer G.-P., de Hosson J.T.M., Brebbia C.A., Nishida S.I. (2007). *In situ* measurement of contact area in coated surfaces. Computer Methods and Experimental Measurements for Surface Effects and Contact Mechanics VIII.

[17] Greenwood J.A., Williamson J.B.P. (1966). Contact of Nominally Flat Surfaces. Proc. Roy. Soc. A.

[18] Bush A.W., Gibson R.D., Thomas T.R. (1975). Elastic Contact of a Rough Surface. Wear.

[19] Jackson R.L., Green I. (2006). A statistical model of elasto-plastic asperity contact between rough surfaces. Tribol. Int..

[20] Požar T., Možina J., Sattler K.D. (2014). Detection of Sub-Nanometer Ultrasonic Displacements. Fundamentals of Picoscience.

[21] Grosse C.U., Ohtsu M. (2008). Acoustic emission testing.

[22] Bobroff N. (1993). Recent advances in displacement measuring interferometry. Meas. Sci. Technol..

[23] Požar T., GregorČiČ P., Možina J. (2011). A precise and wide-dynamic-range displacement-measuring homodyne quadrature laser interferometer. Appl. Phys. B Lasers Opt..

[24] Požar T., GregorČiČ P., Možina J. (2011). Optimization of displacement-measuring quadrature interferometers considering the real properties of optical components. Appl. Opt..

[25] GregorČiČ P., Požar T., Možina J. (2009). Quadrature phase-shift error analysis using a homodyne laser interferometer. Opt. Express.

[26] Křen P., Balling P. (2009). Common path two-wavelength homodyne counting interferometer development. Meas. Sci. Technol..

[27] Dobosz M., Zamiela G. (2012). Interference fringe detection system for distance measuring interferometer. Opt. Laser Technol..

[28] Rerucha S., Buchta Z., Sarbort M., Lazar J., Cip O. (2012). Detection of Interference Phase by Digital Computation of Quadrature Signals in Homodyne Laser Interferometry. Sensors.

[29] Schuldt T., Gohlke M., Kogel H., Spannagel R., Peters A., Johann U., Weise D., Braxmaier C. (2012). Picometre and nanoradian heterodyne interferometry and its application in dilatometry and surface metrology. Meas. Sci. Technol..

[30] Giuliani G., Norgia M., Donati S., Bosch T. (2002). Laser diode self-mixing technique for sensing applications. J. Opt. A Pure. Appl. Opt..

[31] Hoummady M., Farnault E., Yahiro T., Kawakatsu H. (1997). Simultaneous optical detection techniques, interferometry, and optical beam deflection for dynamic mode control of scanning force microscopy. J. Vac. Sci. Technol. B.

[32] Fortunko C.M., Boltz E.S. (1996). Comparison of absolute sensitivity limits of various ultrasonic and vibration transducers. Nondestructive Characterization of Materials VII, Pts 1 and 2.

[33] Heydemann P.L.M. (1981). Determination and correction of quadrature fringe measurement errors in interferometers. Appl. Opt..

[34] Eom T., Kim J., Jeong K. (2001). The dynamic compensation of nonlinearity in a homodyne laser interferometer. Meas. Sci. Technol..

[35] Petrů F., Číp O. (1999). Problems regarding linearity of data of a laser interferometer with a single-frequency laser. Precision Eng..

[36] Požar T., Možina J. (2011). Enhanced ellipse fitting in a two-detector homodyne quadrature laser interferometer. Meas. Sci. Technol..

[37] Lazar J., Číp O., Čízek M., Hrabina J., Buchta Z. (2011). Suppression of Air Refractive Index Variations in High-Resolution Interferometry. Sensors.

[38] Ciddor P.E. (1996). Refractive index of air: New equations for the visible and near infrared. Appl. Opt..

